# EEG-fMRI: Ballistocardiogram Artifact Reduction by Surrogate Method for Improved Source Localization

**DOI:** 10.3389/fnins.2022.842420

**Published:** 2022-03-10

**Authors:** Mateusz Rusiniak, Harald Bornfleth, Jae-Hyun Cho, Tomasz Wolak, Nicole Ille, Patrick Berg, Michael Scherg

**Affiliations:** ^1^Research Department, BESA GmbH, Gräfelfing, Germany; ^2^Bioimaging Research Center, World Hearing Center of the Institute of Physiology and Pathology of Hearing, Warsaw, Poland

**Keywords:** simultaneous EEG and fMRI, artifact removal, optimal basis set (OBS), blind source separation (BSS), multimodal imaging, spatial filter (SF), independent component analysis (ICA), pulse artifact (PA)

## Abstract

For the analysis of simultaneous EEG-fMRI recordings, it is vital to use effective artifact removal tools. This applies in particular to the ballistocardiogram (BCG) artifact which is difficult to remove without distorting signals of interest related to brain activity. Here, we documented the use of surrogate source models to separate the artifact-related signals from brain signals with minimal distortion of the brain activity of interest. The artifact topographies used for surrogate separation were created automatically using principal components analysis (PCA-S) or by manual selection of artifact components utilizing independent components analysis (ICA-S). Using real resting-state data from 55 subjects superimposed with simulated auditory evoked potentials (AEP), both approaches were compared with three established BCG artifact removal methods: Blind Source Separation (BSS), Optimal Basis Set (OBS), and a mixture of both (OBS-ICA). Each method was evaluated for its applicability for ERP and source analysis using the following criteria: the number of events surviving artifact threshold scans, signal-to-noise ratio (SNR), error of source localization, and signal variance explained by the dipolar model. Using these criteria, PCA-S and ICA-S fared best overall, with highly significant differences to the established methods, especially in source localization. The PCA-S approach was also applied to a single subject Berger experiment performed in the MRI scanner. Overall, the removal of BCG artifacts by the surrogate methods provides a substantial improvement for the analysis of simultaneous EEG-fMRI data compared to the established methods.

## Introduction

Interest in simultaneous electroencephalogram (EEG) and functional magnetic resonance imaging (fMRI) experiments has grown, ever since Logothetis et al. (2001) showed a clear relationship between EEG and the blood oxygenation level-dependent (BOLD) signal. Over the years, it has become clear that multimodal data acquisition, in particular EEG-fMRI, provides new insights into neurocognitive functions (Laufs, 2012; Manganas and Bourbakis, 2017). Simultaneous EEG-fMRI recordings benefit from the advantages of both methods—delivering high spatial and temporal precision, and observation of electric and hemodynamic changes at the same time (Mulert and Lemieux, 2010; Rosenkranz and Lemieux, 2010).

The utility of simultaneous EEG-fMRI recordings is limited by three main interconnected factors: (1) the effectiveness of fMRI-related artifact reduction from EEG recording; (2) the usability of analytical tools; (3) the examination cost. Here, effective methods for artifact reduction are of highest importance and a prerequisite for generating useful results. Two types of artifacts are predominant in the EEG signal recorded during fMRI acquisition. The first type is an imaging artifact induced by the magnetic gradient coils (Allen et al., 2000). The second type, the so-called ballistocardiogram (BCG), is related to the heartbeat (Debener et al., 2008) or pulse artifact (Yan et al., 2010). While there is general agreement that the adaptive average subtraction method proposed by Allen et al. (2000) with further improvement from Moosmann et al. (2009) is a sufficient solution for imaging artifact removal, the BCG artifact is still not treated efficiently. The BCG artifact is a complex signal distortion that originates from multiple physical phenomena. As described by the Maxwell equations, the changing magnetic field induces a changing electric field. Therefore, even microscopic head movements in a strong magnetic field generate a strong electrical current. The heartbeat and related blood flow cause whole-body movements when a subject is in supine position (Niazy et al., 2005; Debener et al., 2008). In addition, when an electrode is located near a vein, the skin pulsation can generate another component of artifact (Bonmassar et al., 2002; Yan et al., 2010). Since blood is a conductive fluid, it can generate electrical potential changes over the scalp due to the Hall effect (Müri et al., 1998; Mullinger et al., 2013). Moreover, the BCG artifact can vary over the duration of a recording (Marino et al., 2018a) because of various factors (i.e., position change in MRI, blood pressure change, etc.).

Over the past few years, multiple data processing approaches have been proposed to reduce the BCG artifact (see for review: Grouiller et al., 2007; Vanderperren et al., 2010). Three main trends of BCG artifact reduction can be distinguished: (1) channel-wise subtraction of a BCG artifact template; (2) blind source separation (BSS) based on independent component analysis (ICA); (3) the combination of both methods. The first method evolved from the original work of Allen et al. (1998) and was significantly improved by Niazy et al. (2005). In this approach, the artifact template is created and then subtracted from the data using the optimal basis set (OBS)—the combination of the principal components obtained from the averaged artifact template and the template itself. Further improvements to the OBS were recently proposed by Oh et al. (2014) and Marino et al. (2018b). Most of the changes, however, focus on QRS complex detection and BCG template creation, where the correction procedure is based on artifact signal subtraction which can introduce distortion that mostly manifests itself in topography malformation (Ille et al., 2002). The second approach—Blind Source Separation (BSS)—is of a different nature (Jung et al., 2000). The signal is first decomposed to select artifact-related components. Then, the signal is projected back to the sensor space leaving out these components. The BSS approach usually makes use of ICA algorithms for decomposition as initially proposed by Bénar et al. (2003). Nowadays, there is a vast number of ICA algorithms and many different approaches for component selection (see Vanderperren et al., 2010), which were applied for BCG artifact reduction. Among others, the Infomax (Bell and Sejnowski, 1995; Lee et al., 1999) and the FastICA (Hyvarinen, 1999) have been shown to be successful in reducing the BCG artifact (Infomax: Bénar et al., 2003; Srivastava et al., 2005; Debener et al., 2007, FastICA: Mantini et al., 2007). Nonetheless, the BSS approach could be questioned due to the complex nature of the BCG artifact (Grouiller et al., 2007; Abreu et al., 2016). The independency criterion for ICA might not be fulfilled since the BCG artifact originates from multiple phenomena which result from the same physiological process. One attempt to overcome this limitation is to combine ICA with QRS detection of an EKG electrode either by performing ICA on the epoched data relative to R-peak (Debener et al., 2007) or by clustering approach (Wang et al., 2018). Yet still, the separation between the components of the artifact, as well as the separation of artifact and brain signals, might be insufficient. To address these problems of the mentioned methods, a third approach that is a combination of both methods (OBS-ICA or ICA-OBS) has been proposed (Debener et al., 2007; Abreu et al., 2016; Marino et al., 2018b). Despite the rationality of such an idea, one should consider that the pitfalls of both methods can also propagate to this approach, resulting in high signal distortion when not used carefully (Vanderperren et al., 2010).

To deal with the BCG artifact, there are also hardware-based solutions like reference layer (Chowdhury et al., 2014; Luo et al., 2014) or carbon wire loops (Masterton et al., 2007; Abbott et al., 2015; van der Meer et al., 2016). In those approaches, the BCG artifact is reduced by the subtraction of a referential signal obtained from additional layers/electrodes/loops which record the currents induced by the movement and not the brain activity. It was already shown that this approach can reduce the BCG artifact efficiently (Bullock et al., 2021), however it requires additional hardware and a setup procedure, and also cannot be applied to data already recorded.

In the present paper, we propose a semi-automated BCG artifact reduction method based on surrogate spatial filtering (Berg and Scherg, 1994a). The measured signal is a superposition of brain and artifact activities. In the surrogate method, it is assumed that the artifact signals and the signals originating from the brain can be separated if their spatial distributions over the scalp are known. The artifact topographies can be obtained either by principal component analysis (PCA) performed on an averaged artifact template (Ille et al., 2002) (similarly to the OBS method) or by ICA (like in the BSS method). The brain signals are estimated using a surrogate model consisting of a set of regional dipole sources distributed over the brain to describe most of the EEG signal. Therefore, the BCG reduction procedure should not introduce substantial distortions of the brain signals and separate out the artifact components sufficiently at the same time. In this study, the proposed approaches were compared with the most commonly used OBS, BSS, and OBS-ICA methods described above.

## Materials and Methods

### Subjects

EEG data were collected from 55 young male adults (mean age 27 years). One additional male subject (27 years) was recruited for the Berger experiment. This subject data was part of a previous study (Rusiniak et al., 2018).

All subjects had no history of neuropsychiatric disorders or head injury. Subjects provided written informed consent prior to participation. EEG data processing was performed using BESA Research software (version 7.1 March 2021, BESA GmbH, Gräfelfing, Germany) unless otherwise stated.

### Data Acquisition

Data was collected using an MRI compatible 64-channel EEG system (SynAmps2, Neuroscan, Texas, United States). EEG recordings were performed in a 3 T MR scanner (Magnetom Trio, Siemens, Erlangen, Germany) simultaneously with an fMRI sequence (TR = 3 s, TE = 30 ms, 47 slices, slice thickness = 3 mm, no gap, pixel spacing = 2 × 2 mm). The EEG recording was sampled with 10 kHz frequency starting before the fMRI session and ending after finishing the image acquisition. The EEG sampling clock was synchronized with the MRI machine. Simultaneous EEG-fMRI sessions lasted 6 min (120 volumes). Subjects were asked to observe a black screen (resting-state paradigm) and remain calm. Each fMRI volume acquisition was marked by a trigger event in the EEG data.

A second experiment designed to evoke alpha rhythm in occipital cortex [Berger, 1929 experiment (1929)] was conducted using the same EEG and MRI setup. The subject was asked to open his eyes (when the beep sound was presented) or close them (when the screen was switched to black). Each block lasted for 30 s and the whole recording lasted 6 min.

### Superimposition of Simulated Auditory Evoked Potentials Data

To analyze the efficiency of BCG artifact reduction, simulated auditory evoked potentials (AEP) were added to each resting-state EEG recording using BESA Simulator (version 1.4.0, BESA GmbH, Gräfelfing, Germany). 200 replications of the same simulated AEP signal were added to the original EEG to mimic an auditory EEG-fMRI experiment (inter-stimulus interval = 1.5 s with jitter = 0.2 s) prior to artifact correction or any other signal processing. Two dipoles, oriented perpendicular to the right and left Sylvian fissure, were used to generate the AEP. Source activities were simulated as near-to synchronous mono-phasic Cz-negative deflections (2 ms time lag, parameters detailed in Table 1) and some noise was added with a signal-to-noise ratio (SNR) of 6.

**TABLE 1:** Description of the dipolar model used for auditory ERP simulation.

Source	Location in Talairach coordinates			Orientation			N100 peak
	X	Y	Z	X	Y	Z	Latency
Left	−49	−18	12	−0.17	−0.25	−0.95	101 ms
Right	49	−15	13	0.15	−0.24	−0.96	103 ms

**FIGURE 1:**
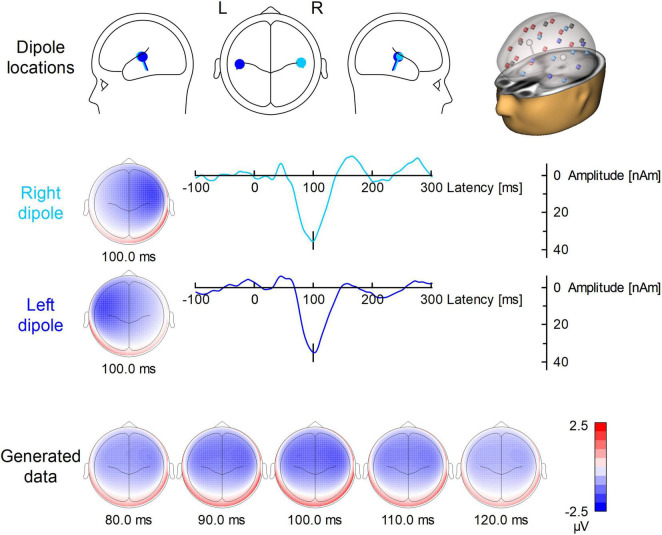
Dipole model used for the simulation of auditory ERP. In the first row, the locations and orientations of the two dipoles are depicted in the head scheme. On the right, the modeled dipoles are shown in the presence of the surrogate model used for BCG artifact correction. The 29 regional sources for surrogate brain model are color-coded (red for right hemisphere, blue for left hemisphere, gray for midline). The modeled sources (which are not part of surrogate brain activity model) are shown in white. The second and third row show the left and right dipole source waveforms along with their topographies. Below, the evolution of the N100 topography from 80 to 120 ms is depicted.

Figure 1 illustrates the locations, waveforms and topographies of the two-dipole AEP simulation (the plots were created using BESA Plot, Version 1.2.3, BESA GmbH, Gräfelfing, Germany). Using this model, the scalp AEP distribution was generated at the 64 recording electrodes and overlapped with the original EEG at the 200 predefined trigger times as specified above. This overlap of AEP and EEG served as the same, identical input for each pipeline of artifact correction to evaluate the differences between methods and to observe the specific distortions of the AEP introduced by each method.

The resulting signal *d*_*k*_(*t*) at electrode *k* can be described as the sum of the measured electrical potential *u*_*k*_(*t*) and simulated *AEP*_*k*_(*t*):


(1) $$d_{k}\,\left(t\right)=u_{k}\,\left(t\right)+A\,E\,P_{k}\,\left(t\right).$$

Since the measured electrical potential *u*_*k*_(*t*) is a mixture of the brain signal *b*_*k*_(*t*), imaging artifact *IMG*_*k*_(*t*), BCG artifact *BCG*_*k*_(*t*), and noise *n*_*k*_(*t*), Eq. 1 can be formulated as follows:


(2) $$d_{k}\,\left(t\right)=b_{k}\,\left(t\right)+I\,M\,G_{k}\,\left(t\right)+B\,C\,G_{k}\,\left(t\right)+n_{k}\,\left(t\right)+A\,E\,P_{k}\,\left(t\right).$$

### Pipeline of Artifact Processing

The pipeline of removing artifacts and retrieving the superimposed, averaged AEP consisted of several steps. First, the imaging artifact *IMG*_*k*_(*t*) was estimated and removed from the data *d*_*k*_(*t*), as described in the fMRI artifact removal section. Second, different BCG artifact reduction approaches were applied to reduce *BCG*_*k*_(*t*). Third, bad trials were rejected, and the accepted *N* AEP trials were averaged as detailed below. The number of rejections depends on the noise level of the EEG, as described in the Evaluation metrics section. Finally, the averaging enhanced the time-locked AEP while minimizing *b*_*k*_(*t*) and *n*_*k*_(*t*). Leaving away the latter terms, this leads to the formula of the averaged AEP:


(3) $${\bar{A\,E\,P}}_{k}\,\left(t\right)=\sum_{n=1}^{N}A\,E\,P_{n,k}\,\left(t\right)$$

$$≅\sum_{n=1}^{N}\left(d_{n,k}\,\left(t\right)-I\,M\,G_{n,k}\,\left(t\right)-B\,C\,G_{n.k}\,\left(t\right)\right).$$

Thus, an optimal IMG and BCG artifact reduction should result in an averaged AEP similar to the simulated AEP.

### Functional Magnetic Resonance Imaging Artifact Removal

The imaging artifact *IMG*_*k*_(*t*) was removed from *d*_*k*_(*t*) by applying the realignment parameter informed moving average artifact subtraction method as described by Moosmann et al. (2009). We used 16 averages as a parameter for moving template creation and a realignment threshold of 0.5 mm. The realignment information was obtained from fMRI preprocessing using Statistical Parametric Mapping software (version SPM12, the Wellcome Centre for Human Neuroimaging, London, United Kingdom) in MATLAB (version 2007, MathWorks, United States). After fMRI artifact removal, EEG data were down-sampled to 1 kHz.

### Ballistocardiogram Artifact Removal

**FIGURE 2:**
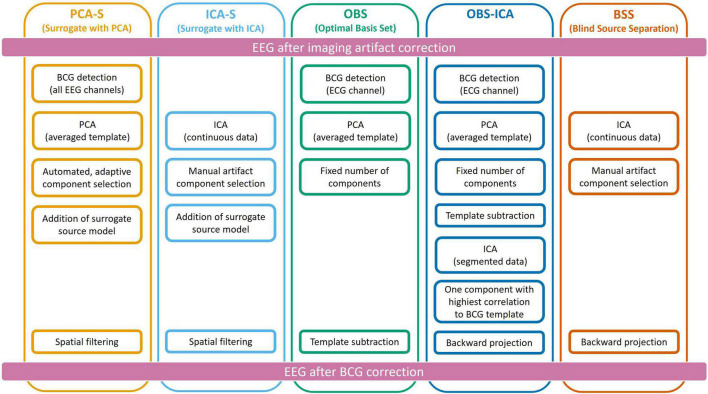
Schematic representation of all five methods used for Ballistocardiogram (BCG) artifact reduction. Data processing was performed from top to bottom, each column represents one method.

To reduce the BCG artifact, five different approaches were used independently as described below and illustrated in Figure 2.

#### Ballistocardiogram Artifact Removal by the PCA Surrogate Method

The PCA Surrogate method (PCA-S) consisted of the following steps: First, for the purpose of creating an averaged template of the artifact, EEG data were band-pass filtered in the frequency range of BCG (1–20 Hz) and re-referenced to the average reference. Then, one representative occurrence of the BCG artifact was manually selected from the EEG data based on visual inspection of all channel waveforms and used for automated pattern search (Scherg et al., 2002; Bast et al., 2004) to create an averaged template of the artifact. Each detected pattern that had a spatio-temporal correlation with the template higher than 60% was accepted. In the next step, a PCA was performed on the averaged template. All principal components accounting for more than 0.5% of the artifact template signal variance were used for spatial filtering. The number of components varied between 4 and 8 (mean 5.7). The accepted artifact-related principal components were combined with predefined regional sources (surrogate model) distributed evenly throughout the brain to calculate a spatial filter that separated the BCG artifact from brain activity as described by Berg and Scherg (1994a). We used a brain surrogate model that included 29 regional sources. A regional source in EEG consists of 3 dipoles at the same location with orthogonal orientations to describe the surrounding brain activity in any direction. Thus, the brain activity was approximated with high goodness-of-fit (>99%) by 87 dipoles (Beniczky et al., 2016). The surrogate model (together with simulated sources which are not part of it) are shown in the first row of Figure 1. By combining the artifact-related principal components and brain-related source components, the inverse spatial filter of PCA-S was created. When applying this linear filter to the original, unfiltered EEG signals, source waveforms with BCG artifact were calculated. Then, data can be projected back onto the scalp EEG using only non-BCG-related data to reconstruct the BCG artifact corrected EEG in sensor space. The brain surrogate model was applied with regularization of 2% and artifact coefficients were applied without regularization.

#### Ballistocardiogram Artifact Removal by ICA Surrogate

The ICA surrogate (ICA-S) method is comparable to the PCA-S method. Instead of using PCA topographies, the BCG artifact components were determined by ICA using the same manual selection as described for BSS (section Ballistocardiogram Artifact Removal by Optimal Basis Set). Then, the spatial components of the ICA traces that were identified as artifact were combined with the 87 spatial brain source components as described for PCA-S (cf. Figure 2).

#### Ballistocardiogram Artifact Removal by Optimal Basis Set

The OBS approach of BCG artifact reduction (Niazy et al., 2005) was used as implemented in the FMRIB plug-in (version 2.0, Nuffield Department of Clinical Neuroscience, Medical Sciences Division, Oxford, United Kingdom) for the EEGLAB toolbox (version 13.6.5.b, Swartz Center for Computational Neuroscience, San Diego, United States). After removing the fMRI imaging artifact, EEG was exported from the BESA Research software into European Data Format (EDF) and loaded into EEGLAB. First, QRS complexes were detected in the ECG channel by the FMRIB plugin (combined algorithms of Christov, 2004; Kim et al., 2004). Second, by averaging the epochs around the detected QRS complexes, an averaged template of the BCG artifact was created. Finally, using the OBS approach, the principal components of the averaged artifact template were subtracted from the EEG. The number of removed components was fixed to 4 which is the default value in the FMRIB plugin. After BCG artifact reduction, EEG data were converted to EDF and reloaded into the BESA Research software for further analysis.

#### Ballistocardiogram Artifact Removal by Optimal Basis Set and ICA

The OBS-ICA method used the outcome of the procedure above (OBS) followed by ICA. This computation was performed in the EEGLAB toolbox following the Debener et al. (2007) processing pipeline. First data was filtered in the range of 0.3–40 Hz and epoched around each detected BCG event (in a range of −50 to −750 ms). Then ICA was computed on concatenated epochs using the Extended Infomax approach (Lee et al., 1999). The component that had the highest spatial correlation with the topography of maximum signal of BCG template was removed during back projection of scalp EEG.

#### Ballistocardiogram Artifact Removal by Blind Source Separation

The BSS approach (Jung et al., 2000) is based on ICA. After filtering with a time-constant filter (low cutoff 0.1 Hz) and a high cutoff filter (30 Hz), a 40 s block of data with clearly visible BCG artifact was selected to perform ICA using the Extended Infomax algorithm (Lee et al., 1999). The largest components were displayed for inspection, and the following visual cues were used to manually identify and mark traces with BCG artifact: waveform shape, and temporal relationship to the electrocardiography (ECG) channel. The number of marked components varied from 3 to 9 (mean 5.5), depending on data. Finally, the BCG artifact corrected scalp EEG was calculated by back projecting only the unmarked ICA components.

### Auditory Evoked Potentials Averaging

Using the artifact-corrected EEG, identical analysis steps were performed for all BCG artifact correction methods. First, bad epochs with residual artifacts like movement or blink were rejected using the automated rejection tool of BESA Research. Epochs with peak-to-peak amplitudes greater than 120 μV and signal gradients greater than 75 μV/sample were excluded. For the detection of bad epochs, data were filtered from 0.3 Hz (forward phase-shift, 6 dB/Oct) to 30 Hz (zero phase-shift, 24 dB/Oct). Second, after rejecting bad epochs, filters were turned off to average the AEP in a window of −300 to +800 ms around the accepted triggers. The averaged signal was filtered using the same filter settings as previously. Finally, EEG data were re-referenced to the average reference of the 64 channels of the artifact-corrected EEG. The grand average AEP was created using the AEPs averages of all subjects.

### Evaluation Metrics

We compared the BCG artifact reduction methods by using the following evaluation criteria: First, for each data correction method that was applied, we compared the number of events that passed the amplitude and gradient acceptance thresholds for averaging. Second, the SNR values of the averaged AEP resulting after applying each method were compared. SNR per channel was computed using the root mean square value of pre-stimulus interval (−300 to 0 ms) as baseline and the root mean square value of the first 300 ms of post-stimulus data as the signal of interest. The mean SNR value across all channels was computed. Third, the averaged AEP waveforms were examined by comparing the latency and amplitude at Cz as detected automatically by the peak detection algorithm of BESA Research in the time range from 0 to 200 ms.

We examined the accuracy of source reconstruction after each BCG artifact reduction method. Since the AEP had been generated by fixed simulated bilateral dipoles (Figure 1 and Table 1), we assessed how much of the averaged signal after BCG correction was explained by the initial AEP model. For this, explained variance was calculated both for the grand average AEP and individual AEP in the full width of half maximum (FWHM) range (81–114 ms). This would amount to 100% if data variance over all channels was fully explained by the model. Lower values indicate higher distortion of the AEP topography.

We also evaluated the location and angle error from single subject source localization. For this purpose we computed a source solution containing two symmetric dipoles for every subject. Dipole locations and orientations were fitted to the artifact corrected averaged ERP using the Nelder-Mead optimization algorithm (Nelder and Mead, 1965) in the range 81–114 ms. A 4-shell ellipsoidal head model was used (the same as for the ERP simulation) (Berg and Scherg, 1994b). The localization error was computed as a norm of difference between obtained and seeded dipole position (c.f. Figure 1 and Table 1). The difference angle was computed as scalar product between dipole orientations. Since in each model there was exactly two dipoles, to simplify further analysis we computed the average error for each pair of dipoles.

None of the tested variables showed normal distribution as tested by Shapiro-Wilk test in the SPSS software (version 21.0, IBM, New York, United States). Therefore, the Kruskal-Wallis (K-W) test was applied followed by Dunn-Bonferroni *post-hoc* pairwise comparison in SPSS.

### Alpha Rhythm Data Analysis (Single Subject)

We compared the eyes-closed state with the eyes-opened state from Berger experiment session using mean fast Fourier transform (FFT). The mean FFT was computed in overlapping blocks (2.05 s) over combined periods of each condition (c.a. 180 s per condition). To investigate the difference, we evaluated the spatial distribution in the alpha range as well as an FFT heat map representing mean amplitude per frequency for each channel, sub-divided into channel groups (frontal, central, left temporal, right temporal, and occipital). In addition, for this data set we compared BCG waveforms from the beginning of the recording with ones from the end of the recording, to evaluate the BCG variability. For this purpose, two different epochs of raw EEG signal after average referencing and filtering (0.3–30 Hz) were sent to the source analysis module of BESA Research. We compared two epochs—one from the eyes-opened state at the beginning of the recording (10 s) and one from the eyes-closed state at the end of the recording (355 s). The epochs were time-locked to the R-peak and the epoch interval was −100 to 600 ms. For both epochs the same model (spatial filtering) was applied, replicating the PCA-S artifact reduction—29 brain regional sources (with 2% regularization) extended with 4 BCG coefficients (with no regularization) obtained from PCA. These were the same components that we used for artifact correction.

## Results

### Mean Trial Number

As an initial measurement of BCG artifact reduction efficiency, we assessed the number of accepted events after rejecting bad epochs. The more accepted events, the higher the quality of the data (fewer residual artifacts).

**FIGURE 3:**
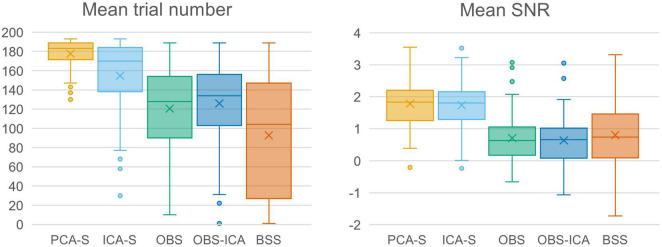
Comparison charts of mean trial number and mean SNR for different BCG artifact reduction methods. Boxes show the medians and 1st and 3rd quartiles, whiskers denote the 1.5 interquartile range, crosses indicate mean values, and outliers are represented by dots.

In Figure 3 (left), the mean numbers of accepted trials were compared. The highest mean value was observed for the PCA-S method ($\bar{x}$ = 178 ± 16). Lower values were obtained for ICA-S ($\bar{x}$ = 155 ± 41), OBS ($\bar{x}$ = 120 ± 44) and OBS-ICA ($\bar{x}$ = 126 ± 45) while the BSS method showed the lowest numbers ($\bar{x}$ = 93 ± 62). There was a statistically significant difference between these methods as determined by K-W test [*H*(4) = 101.1, *p* < 0.001] with a mean rank trial number of 215 for PCA-S, 171 for ICA-S, 106 for OBS, 115 for OBS-ICA and 82 for BSS. *Post-hoc* testing (Table 2) revealed that the higher number of accepted epochs was statistically significant when PCA was compared with all other methods (*p* < 0.001 for OBS, OBS-ICA, BSS and *p* < 0.05 for ICA-S). Similarly, ICA-S outperformed OBS (*p* < 0.001), BSS (*p* < 0.001), and OBS-ICA (*p* < 0.05). There was no significant difference between OBS and OBS-ICA (*p* = 1.000), OBS and BSS (*p* = 1.000) and between OBS-ICA and BSS (*p* = 0.307).

**TABLE 2:** Dunn-Bonferroni *post-hoc* results of pairwise comparison between BCG artifact reduction methods for trial number and SNR.

Pair	Trial number	SNR
PCA-S vs. ICA-S	0.035*	1.000
PCA-S vs. OBS	0.000**	0.000**
PCA-S vs. OBS-ICA	0.000**	0.000**
PCA-S vs. BSS	0.000**	0.000**
ICA-S vs. OBS	0.000**	0.000**
ICA-S vs. OBS-ICA	0.002*	0.000**
ICA-S vs. BSS	0.000**	0.000**
OBS vs. OBS-ICA	1.000	1.000
OBS vs. BSS	1.000	1.000
OBS-ICA vs. BSS	0.307	1.000

Statistically significant values are indicated in bold print, *p < 0.05, **p < 0.001.

### Signal-To-Noise

SNR is a good indicator of the averaged data quality. Higher SNR value indicates a cleaner and less noisy baseline. When the five BCG artifact reduction methods (Figure 3, right) were compared, the highest SNR values were observed for PCA-S ($\bar{x}$ = 3.45 ± 1.60) and ICA-S ($\bar{x}$ = 3.42 ± 1.77). OBS, OBS-ICA and BSS had much smaller values (OBS: $\bar{x}$ = 1.33 ± 3.14, OBS-ICA: $\bar{x}$ = 1.11 ± 3.29, BSS: $\bar{x}$ = 1.43 ± 2.08). The K-W test showed statistically significant differences between these methods [*H*(4) = 81.4, *p* < 0.001]. The mean rank SNR was 193, 188, 100, 96, 112 for PCA-S, ICA-S, OBS, OBS-ICA, and BSS, respectively. *Post-hoc* pairwise comparison (see Table 2) revealed that the higher SNR value observed for both PCA-S and ICA-S was significantly higher (*p* < 0.001) than for OBS, OBS-ICA and BSS. The difference in SNR between PCA-S and ICA-S, as well as between OBS, OBS-ICA, and BSS was not significant (*p* = 1.000).

### Auditory Evoked Potentials Waveform Properties

**FIGURE 4:**
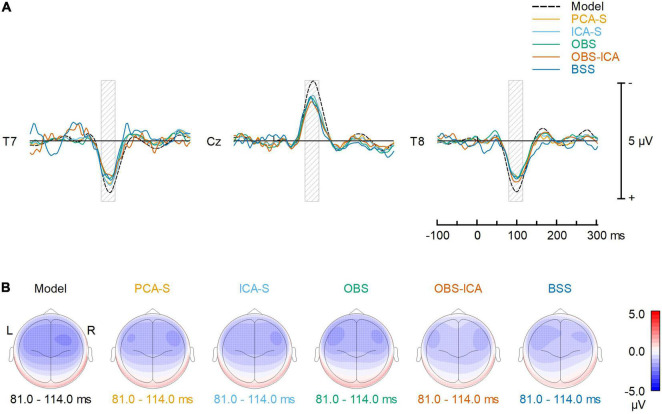
Comparison of the BCG artifact reduction methods. **(A)** The signals recovered at T7, Cz and T8 electrodes (virtually re-referenced to the average reference) are compared to the noise-free, simulated AEP signal. Each solid color line represents one of the 5 different BCG artifact reduction methods; the dashed line (Model) represents the simulated AEP signal. **(B)** Topographic plots of averaged ERP response for the simulated AEP signal and the BCG artifact reduction methods in the range 81–114 ms (full width at half maximum of the modeled signal power, as illustrated by the gray shaded areas in the top row).

To reflect typical ERP evaluation, we compared the averaged AEP signals resulting from the different methods (Figure 4A). The overall waveforms for grand average after BCG artifact reduction were similar to the modeled ones. However, the AEP amplitudes after BCG reduction were slightly reduced as compared to the simulated model for all the methods. Peak latency and amplitude differences between BCG artifact reduction methods were evaluated for N100 at the central electrode (Cz) but no significant differences were found. [K-W test for amplitude: *H*(4) = 3.0, *p* = 0.553, K-W test for latency: *H*(4) = 1.3, *p* = 0.866]. Despite of no difference in amplitude and latency at Cz electrode, some differences in the scalp topography of the grand-mean AEP averaged over the latency range of 81–114 ms were observed (Figure 4B). This could affect source localization which was furtherly evaluated.

### Explained Variance

**FIGURE 5:**
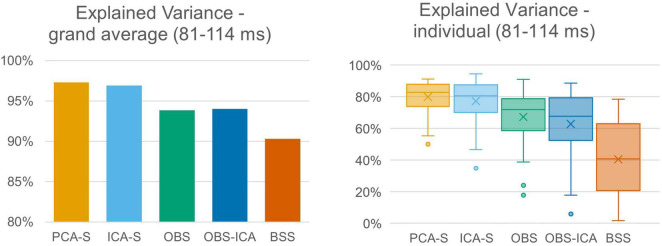
Explained variance of grand averaged data and individual data. Explained variance was averaged across the full width at half maximum of modeled signal power for the different BCG artifact reduction methods. For individual data the boxes show the medians and 1st and 3rd quartiles, whiskers denote the 1.5 interquartile range, crosses indicate mean values, and outliers are represented by dots.

The quality of source reconstruction as defined by the explained variance of the grand average data was highest with PCA-S (97.3%) and ICA-S (96.9%), whereas it was reduced for OBS (93.8%) and OBS-ICA (94.0%), as well as for BSS (90.3%), as shown in Figure 5 (left). Due to the noise of the corrected individual AEPs the mean values of explained variance were lower when considering the mean values over all subjects (Figure 5, right). They were still considerably smaller in OBS ($\bar{x}$ = 67.3% ± 16.0), OBS-ICA ($\bar{x}$ = 62.8% ± 21.1), and BSS ($\bar{x}$ = 40.5% ± 23.8) as compared to PCA-S ($\bar{x}$ = 80.0% ± 9.9) and ICA-S ($\bar{x}$ = 77.3% ± 12.9). The statistical evaluation by the K-W test showed a significant difference between the five BCG reduction methods [*H*(4) = 102.0, *p* < 0.001] with a mean rank explained variance of 194 for PCA-S, 182 for ICA-S, 133 for OBS, 132 for OBS-ICA, and 58 for BSS. The *post-hoc* pairwise comparison is depicted in Table 3. The explained variance obtained for PCA-S was significantly higher than any other non-surrogate-based methods: OBS (*p* < 0.05), OBS-ICA (*p* < 0.001) and BSS (*p* < 0.001). Similarly, ICA-S values were significantly higher in comparison to non-surrogate-based methods, but the difference was slightly smaller (*p* < 0.05 for OBS and OBS-ICA, *p* < 0.001 for BSS). BSS had significantly lower explained variance as compared to both OBS and OBS-ICA (*p* < 0.001). There was no statistical difference between PCA-S and ICA-S and between OBS and OBS-ICA.

**TABLE 3:** Dunn-Bonferroni *post-hoc* results of pairwise comparison between BCG artifact reduction methods for explained variance, localization error and angle error.

Pairwise comparison	Explained variance	Localization error	Angle error
PCA-S vs. ICA-S	1.000	1.000	1.000
PCA-S vs. OBS	0.001*	0.016*	0.040*
PCA-S vs. OBS-ICA	0.000**	0.002*	0.002*
PCA-S vs. BSS	0.000**	0.000**	0.000**
ICA-S vs. OBS	0.013*	0.073	0.264
ICA-S vs. OBS-ICA	0.001*	0.012*	0.026*
ICA-S vs. BSS	0.000**	0.000**	0.000**
OBS vs. OBS-ICA	1.000	1.000	1.000
OBS vs. BSS	0.000**	0.000**	0.000**
OBS-ICA vs. BSS	0.000**	0.001*	0.006*

Statistically significant values are indicated in bold print, *p < 0.05, **p < 0.001.

### Localization Error and Angle Error

**FIGURE 6:**
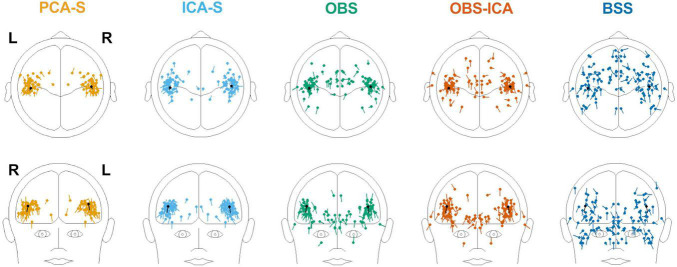
Distribution of source localization obtained by fitting two symmetric dipoles to individual data, for the different BCG artifact reduction methods. In each image the seeded dipole model in the right and left Sylvian fissure is depicted in black.

**FIGURE 7:**
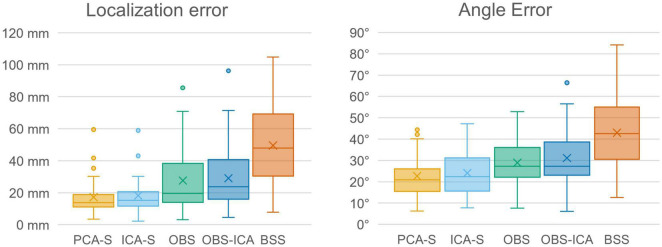
Localization error and angle error for individual model fitting for the different BCG artifact reduction methods. Boxes show the medians and 1st and 3rd quartiles, whiskers denote the 1.5 interquartile range, crosses indicate mean values, and outliers are represented by dots.

Furthermore, we evaluated how the observed difference in explained variance translates to source analysis efficiency. To measure this, we verified the deviation between source model fitted to the artifact corrected data and the seeded model (see Figure 1 and Table 1). As shown in Figure 6, PCA-S and ICA-S had most of the dipoles located around the auditory cortex (where the seeded dipoles were located). For the OBS, and even more so for OBS-ICA, more outliers can be observed. The BSS method resulted in dipoles widely distributed over the whole brain volume. This observation is supported by the numerical verification of localization and angle error, as shown in Figure 7. The localization and angle error were smallest for PCA-S (localization error: $\bar{x}$ = 17.2 mm ± 10.4, angle error: $\bar{x}$ = 22.5^°^ ± 9.8) and ICA-S (localization error: $\bar{x}$ = 18.2 mm ± 11.3, angle error: $\bar{x}$ = 24.0^°^ ± 10.7). A larger error was observed for both OBS (localization error: $\bar{x}$ = 27.6 mm ± 18.4, angle error: $\bar{x}$ = 28.9^°^ ± 11.2) and OBS-ICA (localization error: $\bar{x}$ = 29.0 mm ± 18.5, angle error: $\bar{x}$ = 31.1^°^ ± 11.9). The largest deviation from simulated model was observed for BSS (localization error: $\bar{x}$ = 49.6 mm ± 24.9, angle error: $\bar{x}$ = 43.0^°^ ± 16.3). Further statistical evaluation confirmed that these differences were statistically significant [*H*(4) = 76.2, *p* < 0.001 for localization error, *H*(4) = 63.8, *p* < 0.001 for angle error]. The mean rank values for localization error were 92.3, 99.6, 140.2, 148.8, 209.2 for PCA-S, ICA-S, OBS, OBS-ICA, and BSS, respectively. The mean rank values for angle error were 94.5, 104.5, 138.2, 150.2, 202.6 for PCA-S, ICA-S, OBS, OBS-ICA, and BSS, respectively. The pairwise comparison showed that both localization and angle error for PCA-S was lower than for OBS and OBS-ICA (*p* < 0.05), as well as for BSS (*p* < 0.001). Similarly, ICA-S had lower localization and angle error than OBS-ICA (*p* < 0.05) and BSS (*p* < 0.001). Also, BSS was outperformed by OBS (*p* < 0.001) and by OBS-ICA (*p* < 0.05). There were no statistical differences between PCA-S and ICA-S (*p* = 1.000), ICA-S and OBS (*p* = 0.073 for localization error, *p* = 0.264 for angle error), OBS and OBS-ICA (*p* = 1.000).

### The Evaluation of Single Subject Recording (Berger Experiment)

**FIGURE 8:**
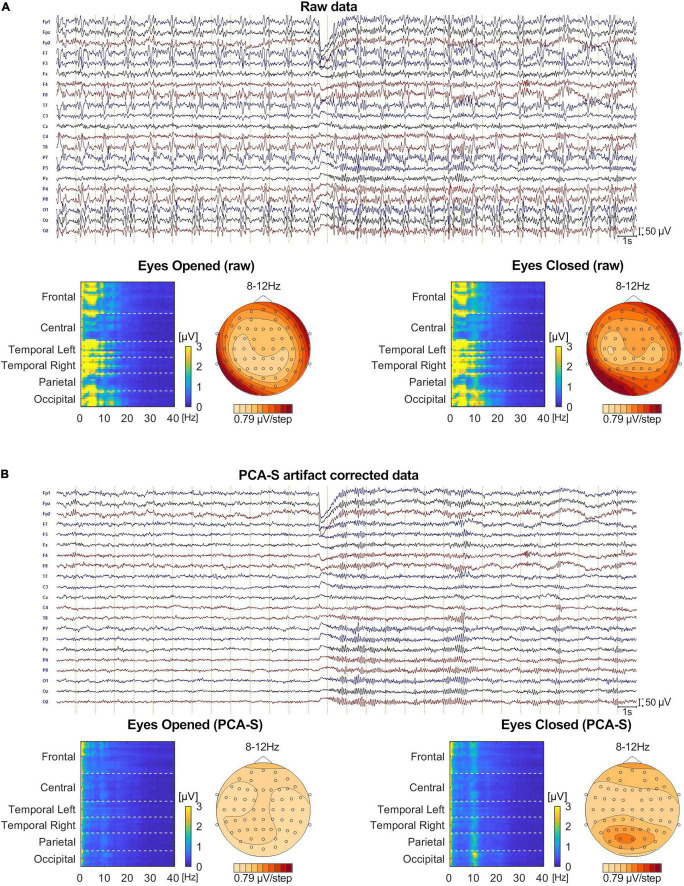
The comparison of Eyes-Opened and Eyes-Closed state for both raw **(A)** and PCA-S artifact corrected data **(B)**. At the top of each sub-figure an example of the same 30 s of recording is shown, in which the transition from eyes-opened to eyes-closed state occurs in the middle. 20 electrodes are shown (every second channel from the 64-channel montage that was used). Below, an FFT heat map for both states, respectively, is shown, showing each recorded channel (grouped by brain lobes) along with the alpha rhythm topography.

No BCG artifact was visible in the data after PCA-S artifact correction, as shown in Figure 8. The blink related to the closing of the eyes is clearly visible in the middle of the shown interval, followed by prominent alpha rhythmical activity. No such activity can be observed before the closing of the eyes. In raw data the blink is also visible, yet due to high contamination with BCG artifact no other data features can be distinguished, even when investigating heat maps and topography maps which are shown below the data interval in Figure 8. Conversely, after correcting data using the PCA-S method, the heat maps depicted a strong differentiation between eyes-opened and eyes-closed states—the activity in the alpha frequency range can be noted, especially in occipital channels. Importantly, in both heat maps, no other atypical oscillatory activity can be observed. The alpha rhythm topography also reflected the normal topography typically observed for the Berger experiment—strong activity in the occipital lobe in eyes-closed state, which is absent during the eyes-opened state.

### Ballistocardiogram Variability Evaluation in a Single Subject Recording (Berger Experiment)

**FIGURE 9:**
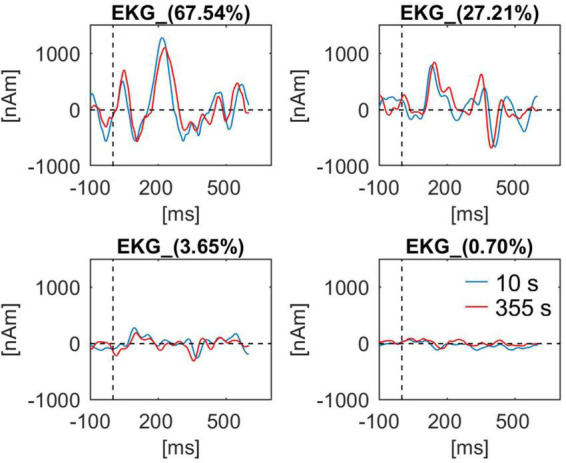
BCG waveforms from a single subject (Berger experiment) for all four artifact components used for artifact correction. Waveforms at the beginning of the recording (10 s, blue line) and at the end of the recording (355 s, red line) are shown. For each component the percentage value of explained variance in the BCG template is indicated in the waveform caption.

In Figure 9 the waveforms for four components of BCG obtained at the beginning (10 s) and at the end of the recording (355 s) are shown. In addition, the first sample was obtained during eyes-opened state, the second during eyes-closed state. While all waveforms are similar, some minor differences can be observed, especially for the second component.

## Discussion

In this study, we applied the spatial filtering method (Berg and Scherg, 1994a) to EEG data measured during fMRI acquisition using a standard surrogate source model in order to reduce the BCG artifact. To compare this approach with the established methods of OBS, OBS-ICA, and BBS, we combined real resting-state EEG data measured during fMRI acquisition with simulated AEPs. Thus, we could evaluate the strength of artifact reduction and signal distortions introduced by the different methods. This approach is justified by the assumption that the fMRI environment introduces only contaminations of the EEG signal and does not influence the brain signals themselves. Importantly, we evaluated our method using auditory ERPs. This bilateral, synchronous activity with tangential dipolar orientation makes the source analysis challenging (Scherg et al., 2019) and highly dependent on SNR. Also, as shown by Shams et al. (2015), the auditory ERPs are more troublesome for BCG artifact correction when compared to e.g., visual ERPs, due to differences in the BCG characteristic across different channels since the generators lie on distant and opposite sites relative to the to head center.

While there was no significant difference between methods for AEP waveform properties, both surrogate-based BCG artifact reduction methods—PCA-S and ICA-S—outperformed the OBS, OBS-ICA, and BSS approaches in the following evaluation metric: the basic signal features (number of events accepted for averaging and SNR), the quality of source reconstruction for the grand average, and source localization error for single subjects. The method used to estimate the artifact topography in the surrogate methods (PCA or ICA) did not have an impact on the AEP outcome, apart from a higher mean trial number accepted for ERP averaging when the PCA method was used.

The number of events accepted for averaging was significantly higher when comparing PCA-S with ICA-S (*p* < 0.05) and OBS, OBS-ICA, BSS (*p* < 0.001, Figure 3, left). A higher number of accepted events for averaging is of major importance since it may lead to shorter experiments and allow for more sophisticated methods of data analysis, for example, the comparison of the first and second part of an experiment, single-trial analysis, time-frequency analysis (Castelhano et al., 2014), or EEG-driven fMRI analysis (Abreu et al., 2018). Furthermore, the higher the number of averaged events, the less biological noise contaminates the waveforms, which can be evaluated using SNR and peak amplitudes.

The highest SNR was observed for PCA-S, followed by ICA-S (Figure 3, right). The significant reduction of SNR in the OBS, OBS-ICA, and BSS methods was mainly due to increased noise introduced before and after the AEP. These findings are in general agreement with the observations of Debener et al. (2007) and explain why the number of detected events was significantly reduced both in OBS and BSS. Interestingly, a slightly lower SNR value was observed when OBS-ICA was compared to OBS, while the number of mean number of trials showed the opposite relationship. This observation stands in contrast to Debener et al. (2007), yet it was not statistically significant here.

Amplitude reduction was observed in all BCG artifact reduction methods, but we did not notice any significant difference between tested methods for single (central) electrode amplitude and latency evaluation. For the first time, this study showed this effect is clearly a product of data processing, as the testing procedure combined modeled AEP activity with real EEG resting state data instead of using test-retest comparisons. However, the cause of this reduction is unclear since it might be due either to the specific BCG artifact reduction process or to the fMRI artifact removal. For simultaneous EEG-fMRI studies, it is widely accepted that signal quality and amplitude is decreased to some extent (i.e., Rusiniak et al., 2013; Marino et al., 2018b). Yet, it is crucial that the MR environment and EEG post-processing do not distort signal topography, in order to minimize the bias of statistical comparisons and source localization. The stability of the signal distribution after BCG correction is also of major importance for a direct comparison of the AEP within and outside of the magnetic resonance device, as well as for longitudinal experiments apart from the documented amplitude reduction. Inspecting topographies obtained on the grand average level for all the methods (Figure 4B) it can be noted that each method affected the topography, yet the outcome of PCA-S and ICA-S reassembled the modeled signal, whereas it looks like the OBS and OBS-ICA introduced some frontal shift in the map. The topography after BSS artifact correction seems to be distorted most.

The differences in topography may likely translate into a distortion of source localization. Therefore, we checked the explained variance of the corrected AEP data when using the simulated AEP source as model. The percentage of explained variance was significantly higher using PCA-S and ICA-S as compared to the OBS, OBS-ICA, and BSS methods. For both surrogate methods the explained variance was around 97%. This value clearly indicates that most of the signal was explained, and that the model is adequate for data explanation. The lower the value, the larger the risk that the model might be considered not sufficient for the data, leading to a perceived need to introduce additional sources to the model. This observation was investigated in more detail by performing analysis on the individual subject level. Obviously, due to much larger noise contamination compared to the grand average, the explained variance at the single subject level was lower in general. The difference between surrogate methods and other methods was even more prominent in this case. For PCA-S as well as for ICA-S, we obtained a mean value around 80% with very low inter-subject variability, while the other methods performed much worse, especially BSS. This indicated the need for a larger number of cases for non-surrogate-based methods to achieve trust-worthy grand average generation. Furthermore, source analysis on a single subject level analysis might not be fully trust-worthy for these methods.

To investigate the reproducibility of single subject source analysis we performed the source fitting procedure on single subjects and evaluated how far from the modeled sources the results were. The source distribution shown for every method in Figure 6, followed by the statistical analysis of localization and angle error shown in Figure 7 and Table 3, clearly indicated that PCA-S, as well as ICA-S provided robust and focused results close to the modeled signal (with mean error values around 20 mm and 20^°^). Most importantly, these methods successfully located activity in the temporal lobe, with just sparse outliers. The OBS and OBS-ICA seem to also lead to correct localization of sources in the vicinity of the modeled sources, yet the high number of outliers as visible in Figure 6 indicate that both methods distort the signal in many cases. The BSS method did not allow for trust-worthy source localization at single subject level at all.

Finally, we verified if the PCA-S method works also for non-ERP data. The Berger experiment is clinically relevant and by far the oldest procedure for evaluation of a non-dipolar, rhythmical signal on continuous data level. In Figure 8 we show that the PCA-S method proposed here allowed for successful BCG artifact reduction and signal evaluation on both visual and computational level (FFT heat maps, alpha rhythm topography). No signal distortion and residual artifacts were observed.

Using this dataset, we also evaluated how PCA-S approach handles BCG variability (Figure 9). Due to changes in cardiac rhythm and blood pressure the artifact changes over time (Oh et al., 2014; Marino et al., 2018a). However, unless the subject changes head position in the MR machine, the spatial distribution is not affected by the aforementioned changes that only impact the temporal aspects of BCG. In this regards the spatial filtering should be unaffected by the physiological artifact variation. The artifact waveforms extracted by spatial filter from data at the beginning and at the end were reviewed. There is a noticeable difference in the waveforms that proved adaptation of data correction to changes in temporal aspects of the artifact. Also, even though first data block was extracted during the eyes-opened state and the second during eyes-closed state, there is no prominent oscillatory activity visible in any of the waveforms. Furthermore, PCA-S, as well as ICA-S, can be easily extended to account for spatial changes by applying regularization to the artifact components, however, that could introduce a risk of data distortion. Here it is worth adding that subject movement in the MR machine is an even bigger issue for fMRI gradient artifact removal, which is based on moving average artifact subtraction, and happens prior to BCG artifact correction in simultaneous EEG-fMRI data processing. Therefore, subject movement should be avoided.

The surrogate approach presented here was already successfully applied in our previous study where simultaneous EEG-fMRI was used for time-frequency analysis of the relationship between alpha rhythm and default mode network in a group of adults (Rusiniak et al., 2018). Recently Plaska et al. (2021, Preprint) applied this approach to evaluate interhemispheric connectivity in working memory during a visual stimulation task.

The classical implementation of the OBS method requires an additional ECG electrode to create the BCG artifact template. Moreover, the OBS method assumes a fixed delay (210 ms) of the BCG artifact relative to the R-wave of the ECG (Niazy et al., 2005). As mentioned before the recent findings show that this value varies with blood transverse time (Oh et al., 2014; Marino et al., 2018a). This problem was partially solved by deriving the triggers for template averaging from the EEG (Marino et al., 2018b), similarly to the creation of the averaged template in PCA-S. In both approaches, the additional ECG channel is no longer required, and the QRS-BCG timing difference is not an issue. However, if no trigger is detected in the OBS-based methods, the BCG artifact will not be subtracted from the signal at this time point. Moreover, in the OBS approach there is no brain signal modeling and artifact components are simply subtracted from the data. In contrast, PCA-S and ICA-S provide a stationary spatial filter (as defined by the inverse separating BCG and surrogate components) that projects out all BCG-artifact components from the EEG at each time point when an artifact occurs. A potential disadvantage of spatial filtering is that a sufficient number of recording channels is needed, amounting to at least the number of artifact and brain source components to enable their separation (Berg and Scherg, 1994a; Scherg et al., 2002). In contrast, a subtraction procedure as used in OBS can be used even for a single channel dataset. However, this limitation is of minor importance in recent studies which use at least 32 EEG channels.

The BSS method uses a stationary spatial filter derived from ICA. The ICA approach implemented here is prone to human error as incorrect components may be selected, or selected components may contain small parts of the ERP activities that are removed with the artifact (Marino et al., 2018a). It is worth mentioning that automatic and semi-automatic approaches do exist (i.e., Srivastava et al., 2005; Mantini et al., 2007; Debener et al., 2008), yet they suffer from the same ICA method limitations. Since ERP signals are typically much smaller than the background EEG, ICA rarely creates independent components for the whole ERP that would be spatially orthogonal to the removed ICA components. Thus, any part of the ERP spatially correlated with the removed components will result in ERP amplitude reduction and distortion if not modeled by another component. In contrast, both PCA-S and ICA-S were specifically designed to remove the correlation between artifact and brain source components, based on the spatial filtering method of Berg and Scherg (1994a). Since the BSS approach is based on ICA and is independent of QRS detection, it acts as a spatial filter on the whole EEG similarly to the surrogate model approaches. However, the BSS approach is fully based on ICA requiring perfect visual selection and separation of the BCG artifact components. An additional problem with ICA-based approaches is the vast amount of possible options for ICA computation (Vanderperren et al., 2010). The BCG artifact is a complex multi-dimensional signal due to the head movement in the strong static magnetic field (translation and rotation in any direction) caused by ballistic forces generated by the heart as well as single electrode movements due to skin pulsation and the Hall effect (Debener et al., 2008; Marino et al., 2018a). It might be scattered over multiple independent components or might not be well separated from another activity. While the manual BCG-related components selection conducted here might be sub-optimal, it is worth to underline that the same selection of ICA artifact components was used in our BSS and ICA-S applications and ICA-S outperformed BSS as well as OBS and OBS-ICA. The fundamental difference of ICA-S is that these components are not simply projected out by the spatial filter (potentially together with some relevant brain activity), but instead they are used as a model of the artifact when separating artifact and brain surrogate components by the inverse filter. Thus, the brain activities are preserved to a large extent in ICA-S as well as by the PCA-S method.

Considering the basic problems of both approaches (OBS and BSS) as shown and discussed here, this combination does not solve the essential problem of subtracting out brain signal together with the artifact in both approaches. While OBS-ICA delivered slightly higher number of mean trial number accepted for averaging, the decrease in all other parameters (SNR, explained variance and localization error) was observed as well. There was no statistical difference between OBS and OBS-ICA but the distortion introduced by the additional ICA step made the difference between OBS-ICA and both surrogate methods (PCA-S and ICA-S) more significant. It is likely that careful manual trimming of OBS and OBS-ICA parameters could improve the efficiency of these methods, but this can be stated for all other methods as well.

BCG artifact reduction is one of the major problems limiting broader usage of simultaneous EEG-fMRI. Taking the above into consideration, the big advantage of the OBS method is its automation and ease of use as implemented in the FMRIB plug-in for the EEGLAB toolbox. As shown here, the PCA-S approach has also been automated apart from the initial selection of one good sample of the BCG artifact. This step could also be automated for future applications (Marino et al., 2018b). Both PCA-S and OBS use PCA to obtain the spatial topographies of the artifact. PCA-S adjusts the number of components using a cutoff criterion based on the variance of each component but does not subtract these components directly from the EEG. In contrast, OBS needs to limit the number of orthogonal PCA components, typically to four, in order not to subtract too much other activity from the EEG due to limited separation from the brain signal.

**FIGURE 10:**
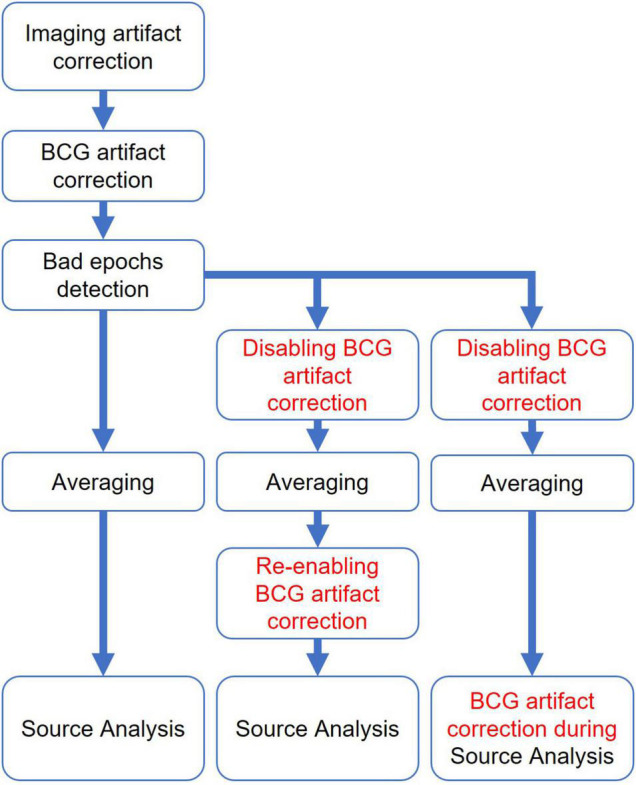
Different BCG artifact reduction pipelines for surrogate artifact reduction methods.

OBS, OBS-ICA, and BSS BCG artifact correction have to be performed prior to the rejection of other artifacts, averaging, and source analysis. Contrary to this, the spatial filter defined by contrasting artifact and surrogate model components is a linear operator which leads to other advantages: It can be applied for the first time prior to the detection of other artifacts and averaging (Figure 10). In addition, the averaging can be done using the uncorrected EEG and the filter is then reapplied to the averaged data prior to source analysis, since averaging and filtering are linear operations and thus commutative (2nd column in Figure 10). The last combination (3rd column in Figure 10) would add the spatial artifact components to the source model of the ERP in order to separate artifact and ERP source activities. Thus, the impact of the number of artifact components and their topographies can be assessed at each stage of data processing in PCA-S and ICA-S and adjusted if required.

In this study, a standard surrogate model of 29 regional sources equally distributed over the brain was used in order to: (a) allow for an automated application of the PCA-S approach and (b) investigate the potential benefit of the surrogate approach even in cases where the source model is not a perfect match of the underlying ERP (e.g., in AEPs), plus for rhythmical non-focal activity. The general surrogate brain activity model might not fit perfectly to every brain data and could lead to some minor distortion, but still the impact of this is less severe than for any other method presented here. Please note that the used surrogate model does not have a source overlap with the seeded model as shown in Figure 1. The adequate choice of an individually generated surrogate model can further improve the rendering of an undistorted ERP when spatial filtering is used for the separation of artifact and brain source components (Berg and Scherg, 1994a). For example, Siniatchkin et al. (2007) used an individual surrogate model of interictal epileptiform discharges recorded outside of the magnet and averaged previously. However, as they pointed out, the limitation of such an approach is to procedures where test-retest can be performed.

While in this paper the focus was on a broad evaluation of source analysis improvement resulting from usage of PCA-S and ICA-S, further assessment of this approach is needed especially for time-frequency and single trial data analysis. The high number of retained events for averaging (Figure 3) might suggest that this approach might be effective for these application types as well. Also, since the surrogate approach is based on spatial filtering and does not need extensive computing it potentially could be used for real-time data processing, after preparing a BCG artifact template during a training phase at the beginning of the recording. Here we performed the evaluation using simulated ERPs superimposed on real EEG-fMRI data. While this allowed for precise assessment of the artifact correction quality there is a need for further evaluation of this approach with real data tests, even though some positive outcomes were already shown (Rusiniak et al., 2018; Plaska et al., 2021, Preprint).

The present study demonstrates that BCG artifact reduction techniques provide more reliable results when surrogate-based spatial filtering is used to correct simultaneous EEG-fMRI recordings especially for source analysis. While for simple ERP evaluation all methods gave similar results, the proposed methods of PCA-S and ICA-S successfully reduced BCG artifacts and preserved the simulated brain signals much better than the established methods of OBS, OBS-ICA, and BBS. This finding was independent of the artifact modeling approach used (PCA or ICA). We also showed that the approach proposed here can be used for evaluation of continuous EEG (Berger experiment) and is unaffected by temporal variation of the BCG artifact. Therefore, the surrogate model approaches can be automated and applied to all types of cognitive EEG-fMRI studies. They have already been implemented in the BESA Research 7.1 software package, and a detailed whole EEG-fMRI pipeline description is available (BESA, 2022).

## Data Availability Statement

The raw data supporting the conclusions of this article will be made available by the authors, without undue reservation.

## Ethics Statement

The studies involving human participants were reviewed and approved by the Ethics Committee of the Institute of Physiology and Pathology of Hearing. The patients/participants provided their written informed consent to participate in this study.

## Author Contributions

MR conceptualized the study, collected funds, performed data analysis, and wrote the first draft of the manuscript. MR, HB, NI, PB, and MS contributed to the methodology. MR, HB, J-HC, NI, and PB worked on algorithms and their implementation. MR and TW collected data and administered the project. MS supervised the project. All authors contributed to manuscript revision, read, and approved the submitted version.

## Conflict of Interest

MR, HB, J-HC, NI, and PB were employees of BESA GmbH, a company which develops and provides software tools for EEG and MEG data analysis. MS was employee and shareholder of BESA GmbH. The remaining author declares that the research was conducted in the absence of any commercial or financial relationships that could be construed as a potential conflict of interest.

## Publisher’s Note

All claims expressed in this article are solely those of the authors and do not necessarily represent those of their affiliated organizations, or those of the publisher, the editors and the reviewers. Any product that may be evaluated in this article, or claim that may be made by its manufacturer, is not guaranteed or endorsed by the publisher.
